# Nonossified cervical vertebrae in Wolf-Hirschhorn Syndrome: A case report

**DOI:** 10.1097/MD.0000000000018268

**Published:** 2019-12-16

**Authors:** You Mi Hong, Dong Hyu Cho, Jin Kyu Kim

**Affiliations:** a Department of Obstetrics and Gynecology, Chonbuk National University School of Medicine; b Department of Pediatrics, Chonbuk National University School of Medicine; c Research Institute of Clinical Medicine of Chonbuk National University–Biomedical Research Institute of Chonbuk National University Hospital, Jeonju, Republic of Korea.

**Keywords:** cervical dysplasia, developmental delay, vertebral bodies, Wolf-Hirschhorn syndrome

## Abstract

**Rationale:**

Wolf-Hirschhorn Syndrome (WHS) is a rare disorder caused by the loss of the distal part of the short arm of chromosome 4, and has various phenotypes depending on the deletion size. Although many articles report on urinary tract malformations or ophthalmologic abnormalities, there are few descriptions of the skeletal anomalies. This is an extremely rare case of cervical dysplasia in WHS.

**Patient concerns:**

A 24-year-old pregnant woman was transferred to our hospital at 21 gestational weeks for intrauterine growth retardation and oligohydramnios and decided to preserve the pregnancy after evaluation. A female was born at full term by normal vaginal delivery, weighing 1791 g. The patient was suspected to have congenital dysplasia of the cervical vertebrae on the routine newborn chest radiograph, and cervical spine magnetic resonance imaging revealed nonossification of the C3 and C4 vertebral bodies.

**Diagnosis:**

The newborn had the “Greek warrior helmet” face typical of WHS. A deletion was detected in the distal portion of the short arm of chromosome 4 (p 16.3) by fluorescence in situ hybridization analysis.

**Interventions:**

She was hospitalized for nutritional management and congenital anomaly evaluation for a month before being discharged with rehabilitation and antiepileptic drugs.

**Outcomes:**

The patient has been readmitted with seizure attacks 5 times to date. At one year of age, she still shows severe head lag and feeding problems. Her last weight was below the 3rd centile.

**Lessons:**

Although cervical dysplasia is a rarely reported morphology in WHS, it may provide artefacts for diagnosing WHS as cervical anomalies, unlike facial anomalies or developmental delays, are seldom found in congenital disease.

## 1 Introduction

Wolf-Hirschhorn syndrome (WHS) is a rare abnormality in which chromosomal material is missing from the short arm of chromosome 4. The sequence of the syndrome was first described by Hirschhorn and Cooper in 1961.^[1]^ In the early 1990s, continued progression in molecular techniques led to the description of the smallest region of overlap of the microdeletions in a number of individuals with the core WHS phenotype, consequently to the boundaries of the WHS critical region (WHSCR) confined as 2Mb terminal deletion of 4p 16.3.^[2,3]^

The incidence of WHS is about 1/50,000 and, presently, >300 cases have been reported in varying degrees of detail.^[2]^ Although this disorder has typical features characterized by the “Greek warrior helmet” face (wide bridge of the nose continuing to the forehead), delayed growth and development, and seizures, it has a broad spectrum of manifestations.^[2,3]^

Skeletal anomalies are found in 60% to 70% of individuals with WHS and include kyphosis/scoliosis with malformed vertebral bodies, fused ribs, clubfeet, and split hands.^[4]^ However, cervical vertebrae abnormalities have been rarely reported in patients with WHS.

## 2 Case presentation

Before the study, we have obtained written informed consent from the patient for publication of the case report and the accompanying images. A 24-year-old woman was transferred to our hospital at 21 gestational weeks for fetal intrauterine growth retardation and oligohydramnios. She was Vietnamese and her husband Korean. She was a multipara and had delivered a healthy boy 4 years earlier. Although her prenatal quad test done at 14 gestational weeks was within the normal range, fetal ultrasonography at this visit revealed extremely low estimated birth weight (303 g, <3rd centile) and a single umbilical artery. We recommended her to undergo an amniocentesis, but she refused and wanted to preserve her pregnancy.

**Figure 1:**
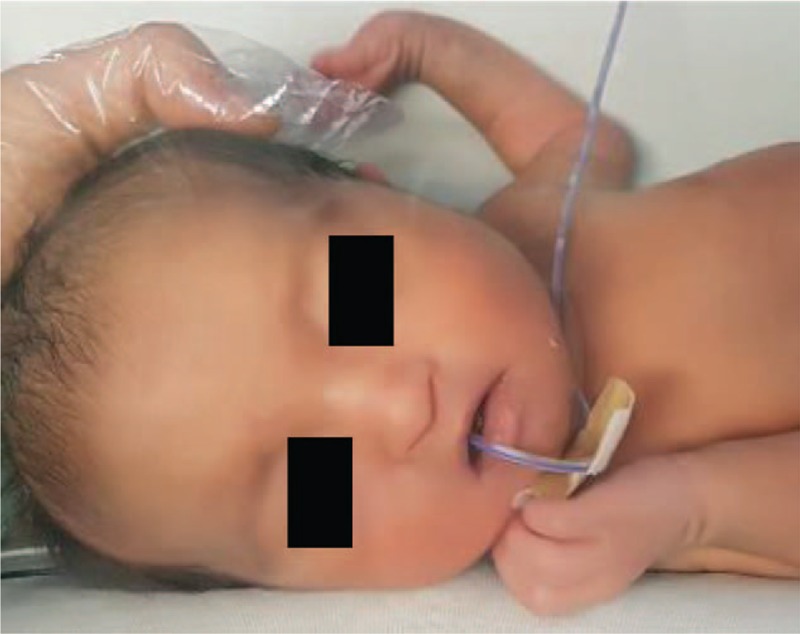
Female patient with Wolf-Hirschhorn syndrome. The patient has “Greek warrior helmet” features (wide bridge of the nose continuing to the forehead) typical for this syndrome.

A female was born at 37 gestational weeks by normal vaginal delivery. The Apgar scores were 7 and 9 at 1 and 5 minutes, respectively. The baby was taken to the neonatal intensive care unit because of her respiratory distress and low activity levels. Her birth weight was 1791 g and length 42.5 cm, the head circumference was 30.5 cm. All measures were below the 3rd centile. As she had dysmorphic facial features such as low-set ears, wide spaced eyes, drooping eyelids, broad-beaked nose, and down turned mouth, she was suspected to have a chromosomal abnormality (Fig. 1). A deletion was detected in the distal portion of the short arm of chromosome 4p by conventional G banded cytogenetic study and FISH analysis confirmed a deletion of chromosome 4p 16.3 region; ish del (4)(p16.3)(WHS-). The karyotypes of her parents were normal.

**Figure 2:**
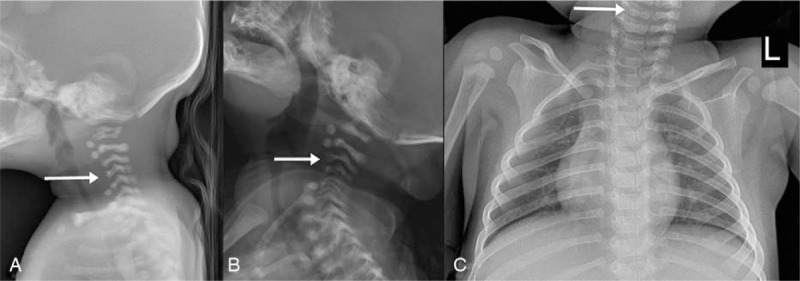
Female patient with Wolf-Hirschhorn syndrome. Lateral cervical spine and AP chest radiograph (A) at birth, (B) at 1 month of age, (C) at 13 months of age. The ossification centers (arrows) of C3 and C4 are invisible in (A) and (B), but visible in (C).

**Figure 3:**
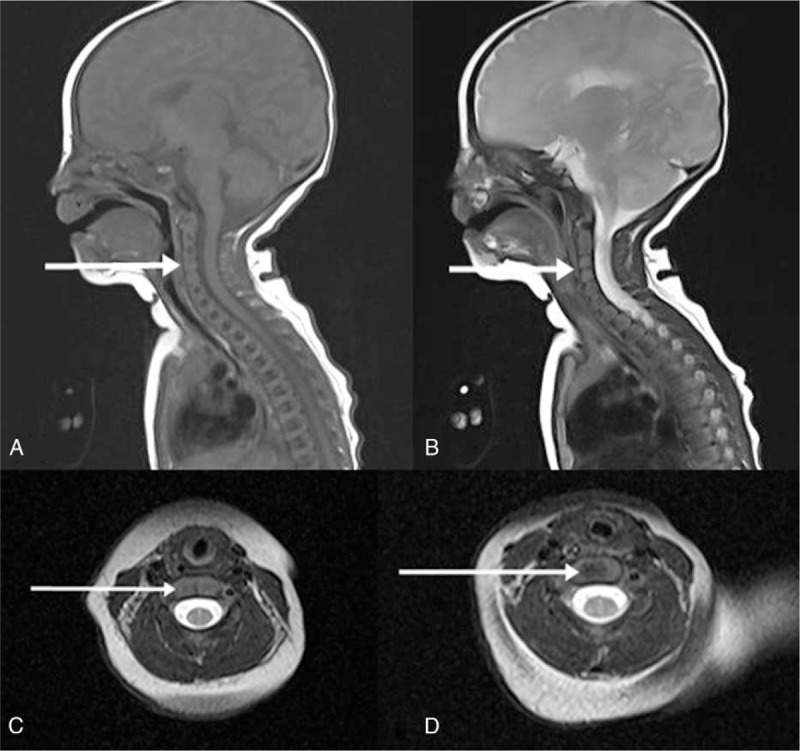
Female patient with Wolf-Hirschhorn syndrome. Cervical spine magnetic resonance imaging at 1 month of age. (A) T1-weighted sagittal view, (B) T2-weighted sagittal view. (C) T2-weighted axial view at C4 level, (D) T2-weighted axial view at C6 level. The hypointense lesions (arrows) in the vertebral body indicate the invisible ossification centers in C3 and C4.

Although the echocardiogram, brain magnetic resonance imaging (MRI), and renal ultrasound all showed normal results, congenital dysplasia of the cervical vertebrae was revealed on the routine newborn chest radiograph (Fig. 2). The ossification centers of the C3 and C4 vertebral bodies could not be visualized on the consecutive cervical spine MRI (Fig. 3). After discharge from the neonatal intensive care unit, she was admitted to hospital repeatedly for her seizures. Although the ossification centers were visible in the follow-up chest radiograph taken at 13 months of age, she still showed severe head lag and other developmental delays. She was feeding poorly and her last weight was 6.1 kg, meaning still below 3rd centile. She has been receiving rehabilitation weekly and is taking antiepileptic drugs.

## 3 Discussion

WHS is a congenital anomaly associated with a partial deletion of the distal short arm of chromosome 4. Wide range of 4p deletion results in variable phenotypes. Three categories of the WHS phenotype can be defined by the size of the deletion. Even though all categories share major features of WHS—such as the characteristic facial appearance, delayed growth and seizures—they can be further distinguished by their specific clinical signs.^[2,3]^

The first WHS category, often called the “mild” form, usually refers to small deletions not exceeding 3.5 Mb. Patients, in this category, can present with different degrees of mental retardation and language fluency, and motor function delay. Major malformations such as congenital heart defects or renal abnormalities are uncommon. The second is the more common category, also referred to as the “classical” form, with the deletion measuring on average 5 to18 Mb. These patients usually present with the typical WHS phenotype, plus severe psychomotor delay, delayed, or absent speech development, and late walking. Major malformations are common, unlike in the first category. The last category is characterized by large deletions exceeding 22 to 25 Mb. In addition to severe psychomotor delay that prevents language and social skills development, the distinct characteristics of this category are facial anomalies that do not resemble the typical WHS appearance. Patients usually can neither sit nor walk without support, and severe scoliosis and psychotic behavior can be additional manifestations.^[5,6]^

It is known that skeletal anomalies are found in 60% to 70% of individuals with WHS, and include kyphosis/scoliosis with malformed vertebral bodies, fused ribs, clubfeet, and split hands.^[2,3]^ Although there are articles reporting various types of skeletal anomalies, most of them focus on appendicular skeletal anomalies rather than axial skeletal anomalies. They are mainly related to nonspecific hand and foot abnormalities including polydactyly, syndactyly, talipes equinovarus, or congenital hip dislocation. There have been only 2 articles that reported on cervical vertebrae abnormalities.^[4]^

One of them was a case series reporting the radiologic findings in 3 WHS patients with underdevelopment of ossification centers in the cervical spine. All patients showed underdevelopment of the neural arches of the lumbosacral spine and underossification of the pubic rami. But other skeletal bone abnormalities were not seen in those patients, particularly no hand or foot deformities.^[7]^ The second article was about a Bosnian female newborn who had hypoplastic C4 and C5 cervical vertebrae and partial agenesis of the corpus callosum.^[8]^

It is uncertain whether the deficient ossification of the cervical spine results from a cartilage anlage or retarded ossification of a normally formed anlage. Each vertebra from C3 to L5 has 3 primary ossification centers, which start appearing at 9 weeks in utero-one in the body and one for each half of the neural arch.^[9,10]^ If the underossification in WHS simply was a result of delayed maturation, progression in ossification would be expected with time, showing normal appearance. In our patient, ossification centers appeared in the C3 and C4 vertebral body at 13 months of age, implying that the phenotype was the result of retarded ossification of a normally formed anlage.

As articles related to the etiology of skeletal anomalies in WHS are rare, reason why the cartilage anlage of the cervical spine is hypoplastic or ossification is delayed in certain patients is not clear. As WHS and other well-recognized autosomal disorders share many clinical features, including skeletal anomalies, we may want to consider and compare with the underlying mechanism in other autosomal disorders. According to an analysis of axial skeletogenesis in autosomal aneuploid fetuses, an uncoordinated morphogenesis of skeletal development is suggested by the delay in ossification of cervical metameres in trisomy 18 and 9, and triploidy.^[10]^ The pleiotropic effect of an unbalanced chromosome set on the prenatal development of the spine extends from neurulation to the late embryonic period.

When patients are seen with underossification of cervical vertebrae, there are several differential diagnostic considerations. For congenital dysplasia of multiple cervical vertebrae, these include the Klippel-Feil syndrome, diastrophic dwarfism, and Larsen syndrome. In Klippel-Feil syndrome, there is an abnormal fusion of ≥2 vertebrae of the cervical spine at birth, resulting in varying degrees of cervical dysplasia. But the disease can be easily differentiated from WHS as it does not have the typical facial anomalies. Differential features of diastrophic dwarfism include progressive dorsal scoliosis and a proximally positioned thumb. Underossification of the cervical spine may occur in neonates with Larsen syndrome, but that syndrome also features characteristic dislocation of multiple joints.^[7]^

Over the recent years, as WHS has been recognized as a multigenetic disorder, a number of the genes related to skeletal anomalies have been studied. There is solid evidence that the WHS core phenotypes, such as distinctive craniofacial features, growth delay, and seizures, are caused by haploinsufficiency of several closely linked genes on the short arm of chromosome 4, including WHSC1, WHSC2, TACC3, FGFR3, and LETM1.^[11]^ As FGFR3 is located in the vicinity of the WHS critical region, homozygous null mouse lines have been created for phenotypic assessment, and these have shown to recapitulate some of the skeletal malformation seen in human patients with WHS. Additionally, mice that are homozygous for a dysmorphic allele of transforming, acidic coiled-coil containing protein 3 have severe defects of the axial skeleton including a complete loss of the caudal vertebrae.^[12]^

We report an extremely rare case of WHS featuring axial skeletal anomaly, that is, cervical vertebral dysplasia. With the recent rapid development of obstetric ultrasonography, the trend of articles on WHS is changing from postnatal diagnosis to prenatal diagnosis.^[13]^ Furthermore, as the majority of fetuses diagnosed with WHS are now being aborted after prenatal testing, less articles reporting unusual features of WHS in newborns are published. Considering that, our report has significance in describing the course of disease in a WHS case with rare clinical features over a year from birth.

## 4 Conclusion

We report a rare case of WHS with cervical vertebral dysplasia resulting from nonossified centers in the C3 and C4 vertebral bodies. It is important that clinicians are vigilant of the possibility of cervical vertebral dysplasia when a WHS patient has neck instability or developmental delay.

## Author contributions

**Conceptualization:** Jin Kyu Kim.

**Investigation:** You Mi Hong, Dong Hyu Cho.

**Methodology:** Jin Kyu Kim.

**Resources:** Dong Hyu Cho.

**Supervision:** Dong Hyu Cho, Jin Kyu Kim.

**Validation:** Jin Kyu Kim.

**Visualization:** You Mi Hong.

**Writing – original draft:** You Mi Hong.

**Writing – review & editing:** Dong Hyu Cho, Jin Kyu Kim.

Jin Kyu Kim orcid: 0000-0002-3502-7604.
